# Improving recognition of patients at risk in a Portuguese general hospital: results from a preliminary study on the early warning score

**DOI:** 10.1186/s12245-014-0022-7

**Published:** 2014-07-10

**Authors:** Nuno Correia, Rui Paulo Rodrigues, Márcia Carvalho Sá, Paula Dias, Luís Lopes, Artur Paiva

**Affiliations:** 1 Emergency Service, University Hospital - Centro Hospitalar São João, Alameda Prof. Hernâni Monteiro, Porto 4200-319, Portugal; 2 Internal Medicine Service, University Hospital - Centro Hospitalar São João, Alameda Prof. Hernâni Monteiro, Porto 4200-319, Portugal; 3 Primary Healthcare Unit ‘Saúde em Família’, Alameda Prof. Hernâni Monteiro, Porto 4200-319, Portugal; 4 Internal Medicine Intermediate Care Unit, Internal Medicine Service, University Hospital - Centro Hospitalar São João, Alameda Prof. Hernâni Monteiro, Porto 4200-319, Portugal; 5 Commission of In-Hospital Resuscitation, University Hospital - Centro Hospitalar São João, Alameda Prof. Hernâni Monteiro, Porto 4200-319, Portugal; 6 Intensive Care Department, University Hospital - Centro Hospitalar São João, Alameda Prof. Hernâni Monteiro, Porto 4200-319, Portugal; 7 Faculty of Medicine, University of Porto, Alameda Prof. Hernâni Monteiro, Porto 4200-319, Portugal

**Keywords:** Emergency, Early warning score, Track and trigger, Triage

## Abstract

**Background:**

Early warning score (EWS) is a system that assists in the timely recognition of hospitalized patients outside critical care areas with potential or established critical illness at risk of deteriorating and who may be receiving suboptimal care. No such systems have been implemented in Portuguese National Health Service's wards. We performed a preliminary study to assess the potential outcome of applying the EWS in our hospital setting.

**Methods:**

An observational retrospective study was conducted based on 100 patients assessed by the outreach team due to an acute event. The EWS was calculated *a posteriori* on three preceding periods from the acute deterioration (−12, −24, and −72 h).

**Results:**

In 35 patients, there was insufficient recording of vital signs. The final sample of 65 patients includes 62.0% men, and the mean age (±SD) was 67 ± 16 years old. Respiratory problems were the main cause of deterioration (44.6%). The EWS score increased from −72 to −12 h. More than half of cases (63.0%) were admitted into high care units, and their mean (±SD) score was higher in comparison to those remaining in general wards (Intermediate Care Units 3.75 ± 1.9, Intensive Care Units 4.2 ± 1.5, wards 3.5 ± 1.4). Score at −24 and −12 h seemed to predict length of stay (LoS; *p* < 0.05) and mortality, respectively. The EWS would have incremented early medical attention by 40.0% if a threshold of ≥3 was used.

**Conclusions:**

EWS systems are not widely used in Portuguese health service. Our data suggests that the EWS would allow early recognition for a higher number of patients in comparison to current ward care. Clinical worsening, lengths of stay, admission into high care units, and mortality may be predicted by the EWS. Prospective studies with multivariable analysis are needed to clarify the global outcome of the EWS implementation in national wards.

## Background

It is clinically intuitive that physiological deterioration precedes critical illness. Several studies have shown that abnormalities in vital signs can help identify clinical deterioration in patients minutes to hours before a serious adverse event occurs [[1]–[7]]. In addition, early intervention has demonstrated to improve patient outcomes [[8],[9]].

Basic observations have been a form of implicit physiological tracking without an explicit trigger [[10]]. Early warning scoring (EWS) systems are tools based on aggregate weighted scoring of physiological variables and provide tracking and a trigger if the total score reaches a predefined alert threshold.

In the UK, many hospitals are using criteria to trigger a rapid medical response based on the modified early warning system [[11]]. In other countries, such as Australia or the USA, ‘calling criteria’ are used to activate a medical emergency team (MET) [[12]].

In Portugal, the General Health Directorate (GHD) recommended in 2010 the implementation of ‘In-Hospital Medical Emergency Teams (MET)’ within an organizational framework that should enable early and rapid recognition and treatment of patients in acute deterioration [[13]]. At the afferent component of the system, MET calling criteria were proposed based on the guidelines of the ‘First Consensus Conference on Medical Emergency Team’ [[14]]. Despite that these criteria may predict an increased risk of death [[12]], they have been considered *late* deterioration criteria [[5]]. On the contrary, a track and trigger system based on an EWS may detect early signs of deterioration [[10]]. These systems have not been widely applied or studied in our national health service.

The GHD recommends that each health care institution should adopt the best strategy to increase clinical monitoring of hospitalized patients [[13]]. Therefore, we performed a preliminary study to evaluate the impact of the EWS system at our setting.

In our hospital organizational model to the critical patient at the time of this study, the on-call medical emergency team was activated according to the established calling criteria in order to assist the deteriorating patient on the wards and decide the need of Intensive Medicine care. After initial evaluation and stabilization of the patient, the MET transported the patient to the Emergency Room (ER) at the Emergency Service, for additional approach, including urgent investigation on the reasons of deterioration, additional treatment, and decision regarding the level of care (return to ward, admission to Intensive or Intermediate Units). The Emergency Room concept in our institution differs from the majority of national hospitals. It is conceived as an extension of the Intensive Medicine care and includes a permanent intensivist physician on 24/7 basis. The assessment of this model of care was not the aim of this study.

Our study's main goals were (1) to assess the EWS in specific time windows preceding the acute event, (2) to study its temporal behavior and its relation with outcomes, and (3) ultimately to compare it with the established ward care.

## Methods

An observational, retrospective, non-controlled study was conducted. The sample consisted of the first consecutive 100 adult ward inpatients assisted by the outreach team and transferred to the Emergency Room, in the period from 1 January to 31 April 2009.

It was assumed that all ward-to-ER transfers were from acutely deteriorated patients who needed additional urgent stabilization. Inclusion criteria are adult patients admitted into the ER from wards of different hospital services. Exclusion criteria are incomplete vital sign records and patients from the Pediatric and Obstetric Services, Intermediate and Intensive Care Units (ICU), and Cardiothoracic Unit.

The studied variables were age and sex; clinical reason for admission at the ER; hospital service of origin of the patient; estimated EWS at −12, −24, and −72 h; in-hospital mortality; and on-call doctor alert. The main clinical reason leading to ward-to-ER transfer was categorized in five groups: ‘respiratory’ (airway or breathing compromise), ‘cardiovascular’ (hemodynamic instability or life-threatening arrhythmias), ‘neurological’ (acute changes in consciousness state), ‘renal’ (urinary output acute changes, metabolic and hydro-electrolytic disturbances), and ‘others’ (all situations not included in the previous items).

Based on records from patients’ clinical files, the EWS was retrospectively calculated at three periods prior to patient ward-to-ER transfer (−72, −24, and −12 h).

The selected EWS system was based on the original EWS developed by Morgan et al. [[15]]. A score threshold of ≥3 was defined as a ‘trigger’ as previously described [[16]–[18]].

Data collection and analysis were carried out with Microsoft Excel 2007®. Categorical variables are presented as absolute and relative frequencies and continuous variables as mean and standard deviation (SD); when relevant, confidence intervals (CI), median, and interquartile range are also presented. A bivariate analysis was based on Student's *t* test to compare continuous variables, including age, length of stay (LoS), and the EWS at −12, −24, and −72 h; *χ*^2^-square test was used to compare categorical variables.

## Results

From the selected 100 patients transferred from the wards to the ER, 18 were excluded due to significant absence of vital sign records (17 were excluded due to errors in clinical archive). Respiratory rate (RR) was the least recorded vital sign. The final sample consisted of 65 patients. Their mean age was 67.7 years old (SD 15.8, minimum 18.0 and maximum 92.0, median 71.0, interquartile range 57.0 to 77.0), and there was higher number of male patients (62.0%).

Before their deterioration and admission into the ER, patients’ mean LoS was 14.4 days (CI 9.2 to 19.6, SD 20.9, median 6.0, minimum 0.0 and maximum 113.0 days). A significant association (*p* = 0.036) between the EWS at −24 h and length of in-hospital stay was observed.

Most of the patients were transferred to the ER during periods of reduced medical physical presence. Nine patients (14.8%) were transferred to the ER in the morning (8:30 a.m. to 1:30 p.m.), 19 (31.1%) during the afternoon (1:30 p.m. to 8:00 p.m.), and 33 (54.1%) at night (8:00 p.m. to 8:00 a.m.). A higher number of patients were transferred to the ER on Fridays (12.0%) and Sundays (23.0%).

Almost half of the patients were transferred to the ER due to respiratory problems (44.6%), followed by cardiovascular and neurological deterioration (27.7% in each group). Patients who deteriorated from respiratory problems came from the Internal Medicine wards (41.3%).

Ward-based physicians and nurses transferred 35.4% of patients to the ER (29.2% and 6.2%, respectively), while 64.4% of the cases were directly managed by the MET.

**Figure 1:**
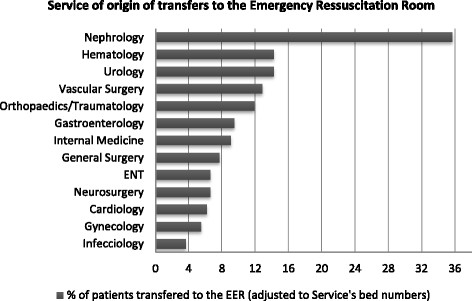
**Proportion of patients’ transfers from wards to ER adjusted to total number of beds of each Service.** ENT, ear, nose, and throat (Otorhinolaryngology Service); %, relative frequency.

Adjusting the number of patients for the stocking of hospital service beds, it was noticed that 47.7% of the patients who deteriorated came from non-medical wards (Figure 1).

Of the 65 patients admitted in the ER, 63.0% were admitted in ICU or Intermediate Care Units (26.0% and 37.0%, respectively), 20.0% returned to their origin wards, and 17.0% died in the ER. The overall in-hospital mortality was 53.8%.

An analysis of the score in the −12 h period revealed that the score was higher, although not statistically significant, in the patients that were admitted into the Intensive or Intermediate Care Units: mean ± standard deviation of 3.5 ± 1.4 at wards (*n* = 12), 3.8 ± 1.9 at the Intermediate Care Unit (*n* = 24), and 4.2 ± 1.5 at ICU (*n* = 17). Retrospective calculation of the EWS on this sample showed an aggravating score tendency on the three evaluated periods before transfer to the ER (Table 1).

**Table 1:** EWS score at three periods preceding ward transfer to the ER

**EWS**	**−72 h**	**−24 h**	**−12 h**
*n*	45	59	65
Mean ± SD	2.6 ± 1.9	2.4 ± 1.8	3.8 ± 1.7
CI (95%)	2.0 to 3.2	1.9 to 2.9	3.4 to 4.2
Median	2.0	3.0	4.0
Minimum	0.0	0.0	0.0
Maximum	7.0	7.0	7.0

**Figure 2:**
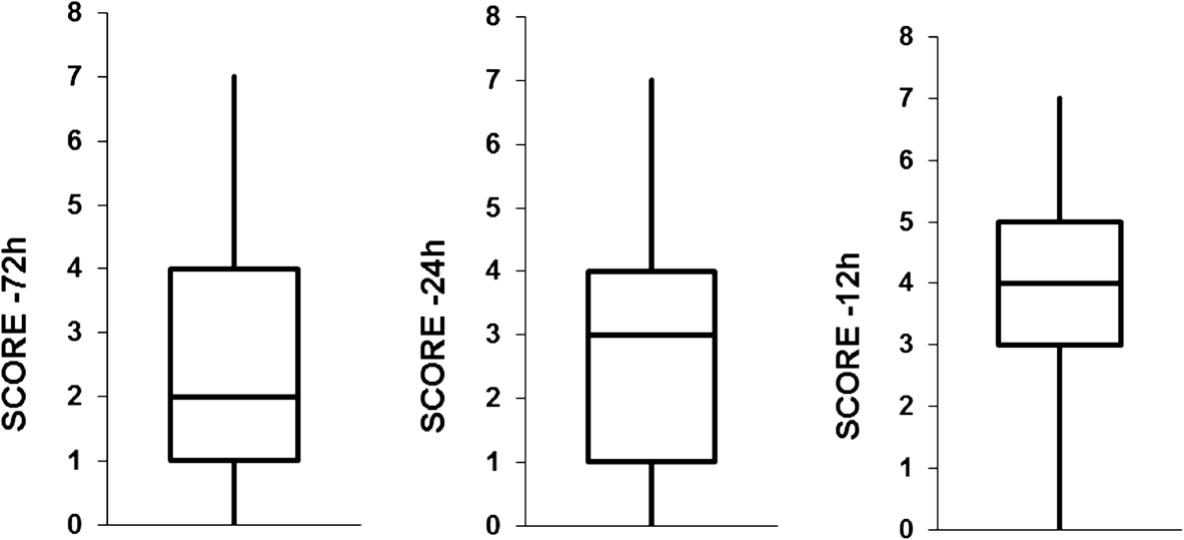
Graphs showing progressive increase in scores toward the moment of acute deterioration.

A progressive increase in scores as approaching to the moment of acute deterioration was documented in three studied periods (Figure 2).

**Figure 3:**
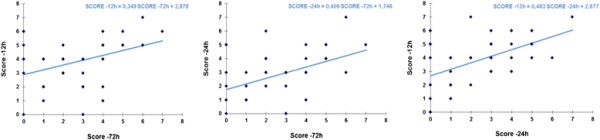
In-between analysis comparing the three periods.

An in-between analysis comparing the three periods (Figure 3) showed a significant correlation between the score at −12 h vs −24 h (*p* < 0.0001), −12 h vs −72 h (*p* = 0.0026), and −24 h vs −72 h (*p* = 0.0007).

Using a threshold of ≥3 on the three periods and comparing it to the occurred alert to the on-call doctor which is based on nurse staff clinical judgment, an increment of alerts based on the EWS ranging from 39.7% to 43.3% would have occurred (Table 2).

**Table 2:** Comparison of real alert of the on-call doctor versus the predicted EWS trigger

**Time window (h)**	**EWS ≥ 3**	**On-call doctor alert**	**Δ (%)**
−72	21/45 (46.7%)	3/43 (7.0%)	39.7
−24	30/59 (50.1%)	4/59 (6.8%)	43.3
−12	50/65 (76.9%)	23/65 (35.4%)	41.5

**Figure 4:**
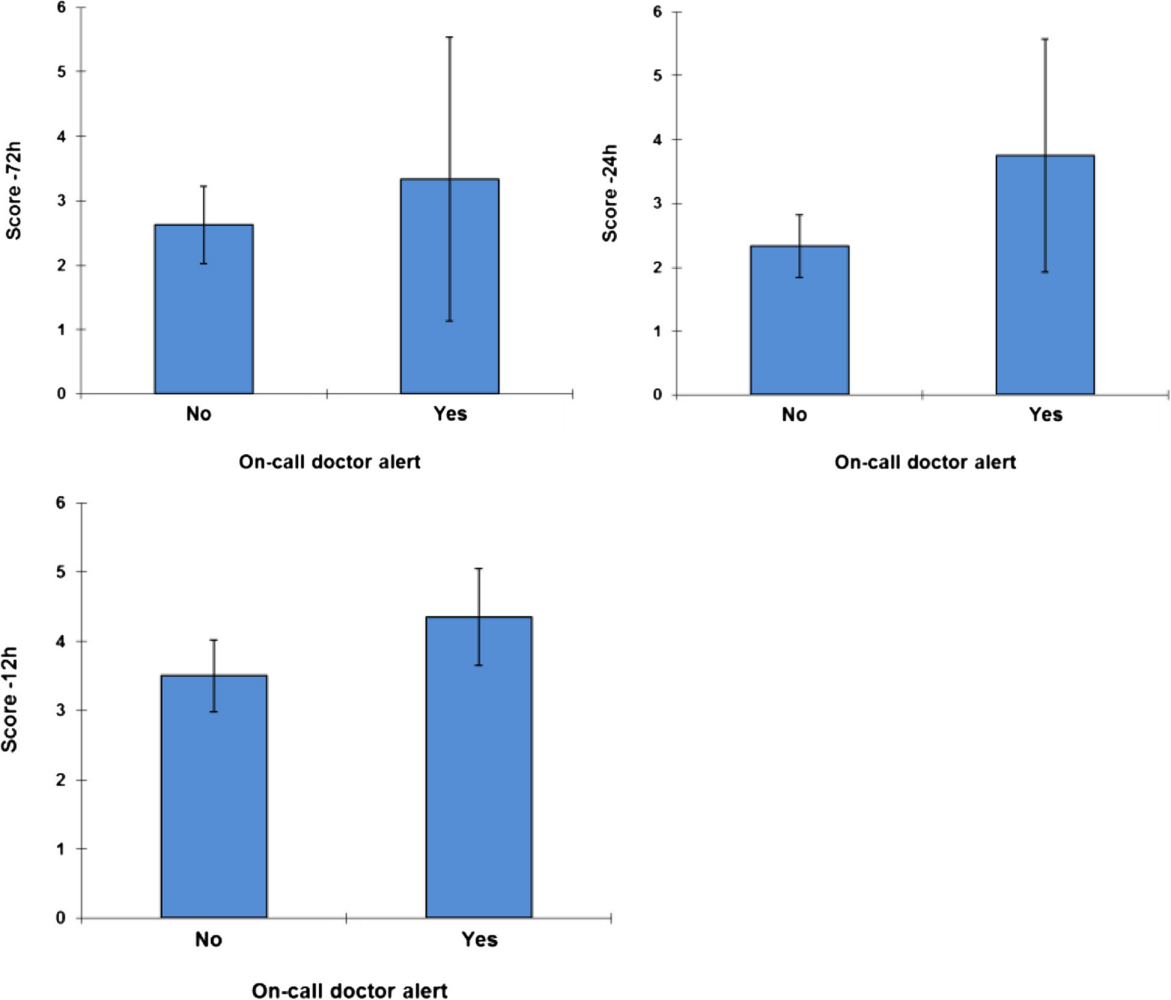
Comparison of EWS between cases in which real alert occurred vs. cases in which no real alert occurred.

Applying the EWS to the set of cases in which a real alert occurred (on-call doctor alert), the analysis shows that all of these patients had a significantly higher EWS in comparison to the group of cases in which no real alert occurred (Figure 4).

Mortality rate was high in the subset of cases with an EWS ≥ 3: 71.4% (15/21) in patients with a high EWS at −72 h, 63.3% (19/30) at −24 h, and 58.0% (29/50) at −12 h. The mean score (±SD) of patients who did not survive after catastrophic deterioration was 4.1 (1.5) at −12 h, 2.5 (1.7) at −24 h, and 2.9 (1.9) at −72 h. In the subset of patients who survive, mean (±SD) EWS was 3.5 (1.9) at −12 h, 2.3 (2.0) at −24 h, and 2.1 (1.7) at −72 h. The EWS score at −12 h was related to the in-hospital mortality, with a mean (±SD) of 4.3 (1.2) in patients who died in the hospital and 3.6 (1.8) in patients who survived, although this difference was not statistically significant.

## Discussion

Safety of the hospitalized patient has been highlighted in the past two decades [[19]]. Patients who are at risk of becoming acutely unwell on general hospital wards receive ‘suboptimal care’ - lack of knowledge regarding the significance of findings relating to dysfunction of airway, breathing, and circulation, causing them to be missed [[20],[21]]. Suboptimal care is enhanced by problems such as not taking vital signs, not communicating concern, and not responding appropriately where physiological deterioration occurs [[19]].

Several studies have shown that vital sign monitoring occurs infrequently and their measurements may not be performed predictably, accurately, or completely [[22]–[25]]. In our sample, records on vital signs were missing in almost one fifth of the patients. Respiratory rate, an early indicator of disease [[24],[26]], was very often neglected by the ward staff, a finding that has already been noticed by others [[24]]. Importantly, the use of an EWS has shown to improve ‘observations’ records [[27]].

We have only found one study with EWS in Portuguese national hospitals. A prospective ‘action-research’ study in 113 surgical patients in 2009 found that nearly 20% presented a risk score (>3) which leads to activation of medical assistance in 33% of their EWS records. It was found that RR was consistently less valued, although it was the vital sign that contributed the most to final score changes. The authors concluded that an EWS in association with a medical activation algorithm translates into benefits for patients as well as ward staff [[28]].

Our study has limitations due to its retrospective methodology, the small sample size, and lack of multivariable analysis. However, it was able to provide preliminary relevant data.

Of note, we did not exclude patients in palliative care or under ‘do not resuscitate’ orders. In agreement with other authors [[16]], we believe these patients demand a sensible management that should implicate both patient (if possible) and relatives. An EWS might help in early definition of their ‘optimal’ care.

Our research revealed that most acutely deteriorated patients were elderly or older adults, with 50.0% of them aging between 57 to 77 years. Older age, although not included in patient assessment by EWS, may influence patients’ resilience and predisposition for catastrophic deterioration. A relation of age and mortality with higher EWS scores has been demonstrated, and it was suggested that inclusion of age in EWS could be advantageous in improving EWS function [[29]].

Several studies have demonstrated an increase in in-hospital mortality at night or during weekends [[30],[31]]. These ‘weekend effect’ was also observed in Portuguese hospitals [[32]].

Our data shows a higher number of transfers from wards to ER during periods of less medical attendance (afternoon and night) and weekends. Although it might be a consequence of random effect, our local perception is that patients’ deterioration is high during these periods. This may be explained by a lack of awareness on patients’ impending deterioration by the ward staff, problems in shift handovers, and periods of diminished physician attendance as a consequence of local working organization.

Our study showed that patients deteriorate mainly due to respiratory problems and that most of them stay in the Internal Medicine Service. Two main reasons may contribute to this figure: (a) in our setting, the majority of patients in Internal Medicine wards suffer from chronic cardiorespiratory diseases, and (b) an airway or breathing problem may be easier to recognize by nurse staff. These results reflect the nature of the organization models of Portuguese hospitals.

Non-medical wards were responsible for almost 50% of admissions in the ER. This finding may be explained not only by a greater physical fragility of surgical patients, but also by the lower number of physicians assigned to these wards in comparison to medical wards. The EWS system would be helpful in assisting health care staff in these wards, a benefit that has already been demonstrated [[33]].

Despite the research on EWS outcomes, the original EWS was developed not as a predictor of outcomes but solely as a tool to ‘secure the timely presence of skilled clinical help by the bedside of those patients exhibiting physiological signs compatible with established or impending critical illness’ [[10]]. We share the view of Morgan et al. that the clinical course of a critically ill patient is influenced by a multitude of factors that trying to predict outcomes on the basis of routine observations may be an ‘unrealistic expectation’ [[10]]. This complexity may explain why several studies suggest that EWS may predict outcomes [[16]–[18],[29]] while others found no impact in patient outcomes [[34],[35]]. Nevertheless, we have found interesting associations between EWS and patient's outcomes.

The mean hospital stay before catastrophic deterioration was 14.4 days, and we only found a correlation between LoS and EWS at −24 h which may be due to the small sample size. Groarke et al. observed a correlation between higher EWS on admission and LoS [[18]].

More than half (63%) of the ‘acutely ill’ patients on the wards ended up admitted to a higher level of care (Intermediate or ICU). Our analysis suggests that patients admitted into these units had a higher score in comparison to patients that returned to the wards. Subbe et al. prospectively demonstrated an association between a raised EWS and increased mortality and admission into ICU and high-dependency units [[16]].

Our data demonstrates that EWS correlates with patients’ deterioration (Figures 2 and 3). This finding suggests that EWS may predict patients at greater risk and in need of more medical intervention.

There was also a relation, although non-significant, between EWS and mortality at the ER as well as in-hospital mortality which emphasizes the potential of this score in predicting outcomes. More than half of patients admitted to the ER have ultimately died during the hospitalization. At a time of limited resources, a low cost and easy to apply system, such as the EWS, would allow carefully planning the admission and optimizing the safety of patients in general wards. Therefore, any improvement in the prevention of catastrophic deterioration would probably have a beneficial impact. Nevertheless, a prospective study, with a comparative arm, is needed to evaluate the outcome of the EWS.

Among patients who have died after admission into the ER, the EWS seemed to be higher on the preceding periods, with the greatest score difference on the ‘−12 h’. This result highlights the potential of the EWS in predicting catastrophic deterioration. Several studies have already concluded that an EWS can identify patients in need of hospital admission, predict morbidity and mortality, and diminish ‘code blue’ events as well as admissions into the ICU [[17],[18],[36],[37]].

Based on the EWS, different trigger cutoffs and physiological values have been used without prospective validation, raising problems on sensitivity and specificity of the system [[16],[38]]. Duckitt et al. validated a scoring system after a multivariate logistic regression analysis in a large sample of patients admitted to the Emergency Care Unit. After comparison of the new derived system with the EWS as recommended by the UK's Department of Health, it was found that the cutoff point that gave maximum sensitivity and specificity was 3 (sensitivity and specificity of 0.63 and 0.72 vs 0.60 and 0.67, respectively) [[39]].

Using a trigger threshold of ≥ 3, we calculated an increment of nearly 40% of alerts in comparison to current ward care. This level would lower to approximately 20% if the threshold was raised to ≥ 4. These variations could lead to different outcomes regarding patients’ care. Therefore, the optimal trigger score deserves additional research in order to prevent excessive evaluation of patients who have abnormal vital signs but who are not at risk for serious adverse events and to avoid failures in recognition of patients potentially ‘at risk’ [[12]]. Whatever the trigger cutoff and physiological variables, the increase in medical workload must be accepted [[40]] since the EWS improves care quality in comparison to current ward care and clinical judgment.

## Conclusions

In Portugal, track and trigger systems are not widely applied. The outreach system afferent component has relied on ‘MET calling criteria’ which is considered a *late* recognition system of patients at risk.

Our results revealed that the EWS objectively correlates with patients’ impending deterioration and may predict admission into higher level of care units, length of stay, and in-hospital mortality. In comparison to current clinical ward care, the EWS would have significantly increased the detection of critical ill patients by ward staff by 40%. This enhancement in surveillance would probably yield a huge benefit on patient outcome since the current in-hospital mortality of patients at risk is very high (around 50%).

The available literature suggests that the benefit of the EWS outweighs the predicted increase in medical workload. Nevertheless, more prospective research on EWS systems is needed to establish the appropriate variables, the most sensitive and specific warning threshold, as well as its effect on patients’ outcomes.

The EWS may be a valuable auxiliary tool to assess patients’ risk of deterioration at the ward level. Allied to periodic reinforcements in staff education and organization, systems such as EWS may bring us closer to the ‘optimal care’.

## Competing interests

The authors declare that they have no competing interests.

## Authors’ contributions

All authors have made substantial contributions to the paper. NC and LL were involved in study conception. NC gathered all the data and drafted the main manuscript. RR and MS actively cooperated in statistical analysis and actively contributed to result interpretation and review of the manuscript. LL additionally contributed with final remarks. PD made important suggestions on the interpretation of results and general review. Professor AP made substantial contributions in revising the paper critically for important intellectual content and scientific relevance. All authors approved the final version to be submitted. We declare that we had no writing assistance. All manuscript's data, figures and tables, have not been published previously, and the manuscript is not under consideration elsewhere. The authors suggest following two reviewers who have not been involved in the design, performance, and discussion of the data and are not co-workers.

## Setting

The work was performed at the University Hospital São João, in Porto, Portugal.
